# Beyond Neutrality—Ecology Finds Its Niche

**DOI:** 10.1371/journal.pbio.0040278

**Published:** 2006-08-15

**Authors:** Virginia Gewin

From physics to ecology, one formidable goal of scientific exploration is determining the forces at work in nature and how these forces organize our world. In trying to uncover simple laws, scientists must balance the accuracy and complexity necessary to describe essential mechanisms. Sir Isaac Newton's laws of motion were sufficient for almost 200 years, but Einstein's addition of a fourth dimension of space-time was justifiable because it not only increased the accuracy and complexity of understanding but also moved physics past a descriptive stage. Understanding the patterns of biodiversity in a tropical forest or a coral reef, however, has had ecologists mired in the multiple dimensions of natural laws to simply describe how species survive and co-exist. Distilling this complexity to the essential drivers of species assemblages will not only help ecology meet its most daunting conservation challenge—staving biodiversity loss—but also help move the science into a predictive stage.

It seems intuitive that every species should have its niche. Polar bears live in wide spartan territories of the Arctic, while earthworms live in small, organically rich patches of earth. Under a canopy of tall, sun-seeking trees live shade-tolerant plants. One hundred years of observation has formalized this concept of the niche. Through natural selection, species adapt physically, physiologically, and behaviorally to their surroundings while carving out their unique position in an ecosystem. Niche theories, which are many and varied, have focused primarily on trade-offs, such as competitive ability within a particular environment, to explain the abundance and distribution of species. But, to resolve more generally why communities often have many rare species, and only a few abundant ones, niche theories based solely on such species-determined traits have not yet sufficed.

“Other theories don't suffer the ignominy of having a self-destruct button.”

Enter the reductionists. Unconvinced that species differences alone drive community dynamics, ecologists Stephen Hubbell at the University of Georgia in Athens, Georgia, United States, and Graham Bell at McGill University in Montreal, Canada, independently developed a theory to determine the extent to which patterns could be explained by random, or stochastic, forces beyond a species' control. Throwing out traits such as competitive advantage entirely, and, even more heretically, viewing different species as functionally equivalent, Hubbell's controversial *The Unified Neutral Theory of Biodiversity and Biogeography* relies on nothing more than randomness of births, deaths, speciation, and dispersal to describe the distribution of species in an environment. Surprisingly, given its extreme simplification of seemingly complex phenomena, this neutral theory successfully describes the observed species abundance patterns of numerous communities.

Despite the fact that more stringent tests of neutral theory's predictions have not held up well recently, its systematic approach has forced ecologists to explore its tenets. In fact, many ecologists now accept that the theories are not mutually exclusive. By dissecting the degree to which random events shape the biodiversity of ecosystems, the importance of more deterministic processes of natural selection can be evaluated. As ecologists explore the combination of species-determined and random mechanisms capable of predicting species abundance patterns, some predict a future merger of the two theories. In doing so, ecologists are striving for a degree of predictive power that will demand a level of rigor as yet unseen in ecology.

## Neutral Theory Beats the Odds

**Figure 1:**
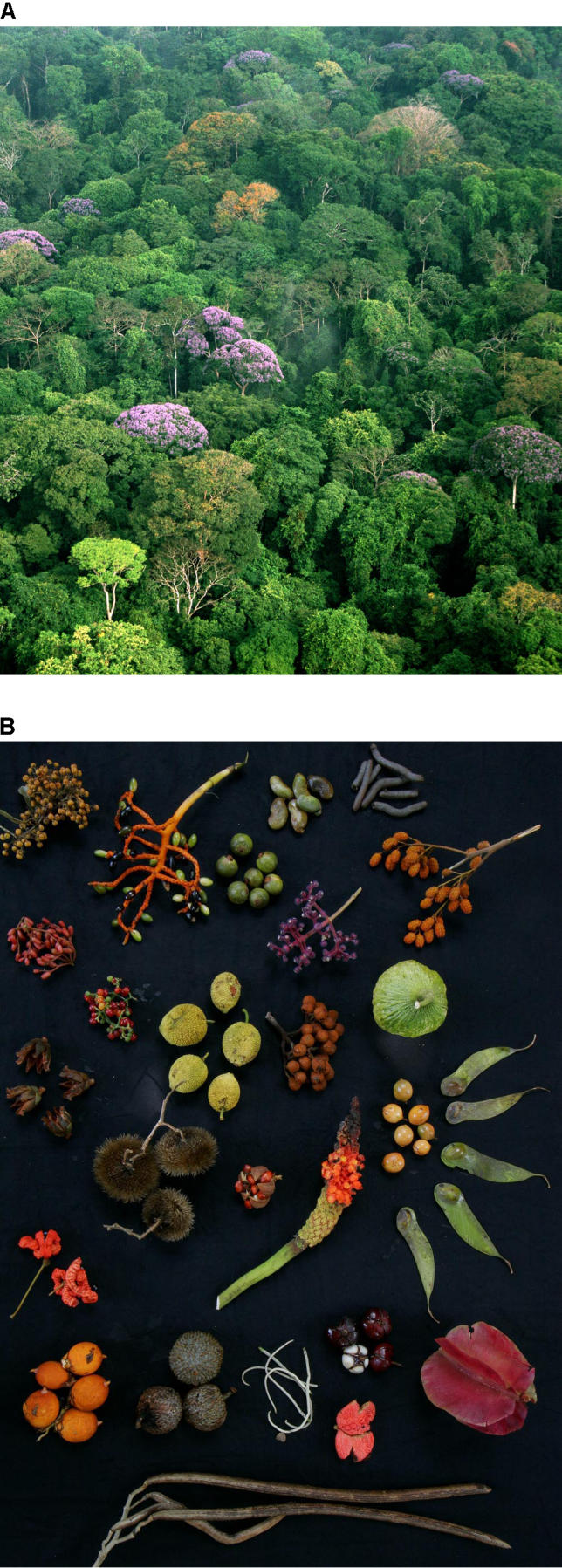
Conservation Efforts Hinge on Understanding the Factors Controlling Biodiversity (A) The diverse forest canopy on Barro Colorado Island, Panama, has provided ecologist Stephen Hubbell with years of data to test his controversial neutral theory of biodiversity and biogeography. (B) Despite the different fruit types and dispersal modes pictured here, Hubbell wonders how well patterns of diversity can be explained by focusing on the similarity of species rather than their differences. Photos courtesy of Christian Ziegler.

Neutral theory has its roots in population genetics (see Box 1), which also relies on the notion of randomly generated diversity. In ecology, two mechanisms regulating diversity have come to the fore. First, Hubbell showed empirically that dispersion (i.e., the spatial distribution of plants and other organisms) is not unlimited. Specifically, in a study of more than 300 tropical forest trees and shrub species present on Barro Colorado Island of Panama, he found that the majority were somehow prevented from occupying suitable habitat. During a ten-year period, only 12 species made it into at least five of the 200 seed traps located throughout the island. From this, he incorporated limits of dispersion into neutral theory (Figure 1).

“Anyone interested in determining what controls species abundance patterns has to come up with an equally simple theory, or a more complex theory that made better predictions.”

Neutral theory has also recently been modified to reflect increasing evidence that birth and death rates are not fixed, but instead depend upon population density. For example, a large population of one species is more susceptible to predators or pathogens. Rare species can therefore have an advantage simply because of their rarity. While some hypothesize that asynchronous reproduction and mortality keep abundant species in check, those same processes also afford rarer species the opportunity to exploit resources. The importance of dispersion and density dependency helped root neutral theory.

However, the power of neutral theory in ecology lies not in its rules, but in its violations. Even its principal assumption—that species are functionally equivalent—has been proven false by increasingly rigorous tests. Consequently, with the theory as a backdrop, important species-specific traits are being uncovered. It offers, in essence, a null-hypothesis: if the data can be explained by neutral theory, then they require no other specific traits. “Any interesting ecologically relevant mechanisms now have to be tested against neutral theory,” says Jerome Chave, ecologist at the Universite Paul Sabatier in Toulouse, France. Using Hubbell's data from the tropical forests on Barro Colorado Island in Panama, one recent paper noted that the diversity of the rainforest increases with tree age. The findings suggest that random forces alone aren't responsible for the patterns seen; active selection processes must be at work. In addition, Brian McGill, theoretical ecologist at McGill University, used fossil records to show that mammalian communities changed less during the past million years than would be predicted by neutral theory, suggesting that particular community is naturally selected for time and again.

Hubbell sees such so-called failures of neutral theory as triumphs. “The fact that it gets rejected is precisely because we built in a way to reject it,” he says. “Other theories don't suffer the ignominy of having a self-destruct button.” Indeed, when neutral theory fails, it often identifies an important underlying mechanism at work.

**Figure 2:**
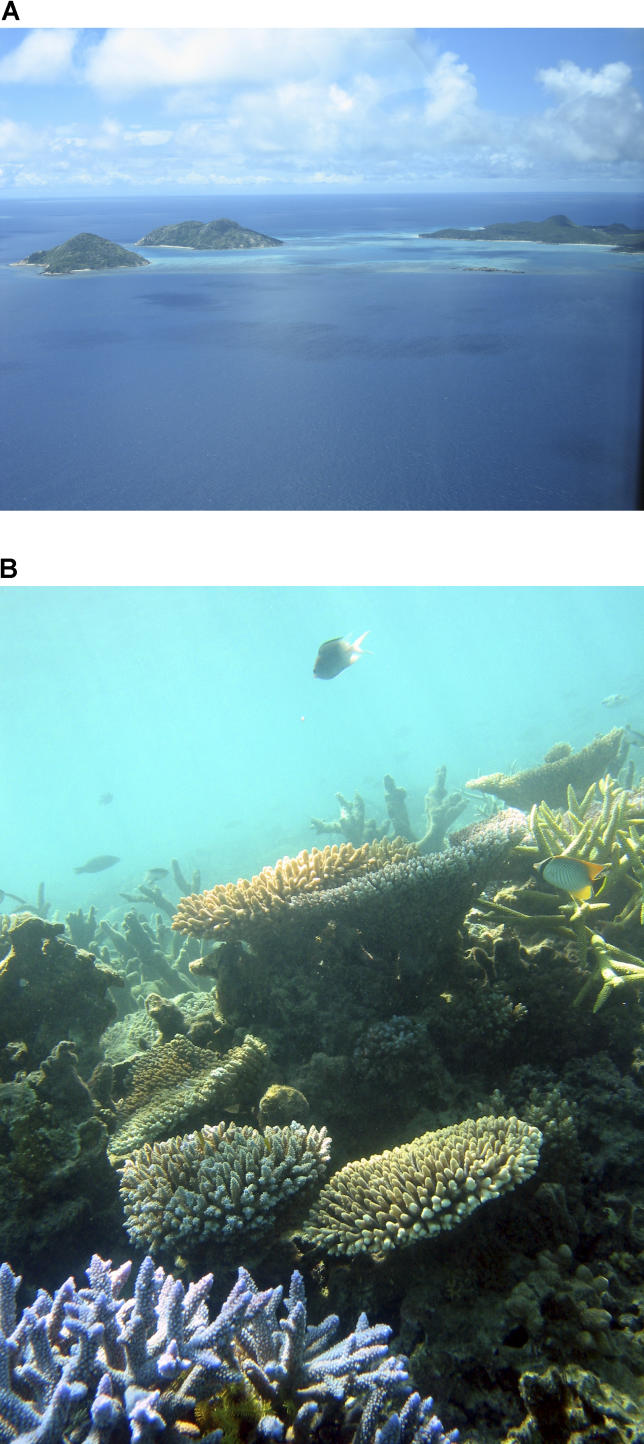
Diverse Coral Reef Systems Serve As Ideal Experiments for Niche and Neutral Theories (A) Surveys of coral reefs on the Great Barrier Reef in Australia, and elsewhere in the Indo-Pacific, are prompting both niche and neutral theorists to pay greater attention to the role of environmental fluctuations on species diversity patterns. Photo courtesy of Sean Connolly. (B) Organisms cover every square inch of coral reef, which led many to believe that their limited potential to partition resources into niches would make them a prime example of neutral dynamics. In fact, species diversity was more variable than would be assumed by neutral theory. Photo courtesy of Terry Hughes.

For example, coral reefs were thought to be the best example of neutral dynamics. They are highly diverse with a limited potential to partition resources into niches. But when coral reefs were surveyed along a transect from the Indian and Pacific oceans, they were more variable than the demographic randomness predicted by neutral theory (Figure 2). The authors suggest this is due to species varying responses to environmental fluctuations. “If species are identical, they shouldn't respond in different ways to environmental fluctuations,” says Sean Connolly, ecologist at the ARC Centre of Excellence for Coral Reef Studies and School of Marine Biology and Aquaculture at James Cook University, Townsville, Australia, and co-author of the coral reef paper. “The role of environmental fluctuations and variability has largely been neglected,” he adds. Indeed, Hubbell's work has focused on random demographic mechanisms, not the forces at play in the environment—which many niche theorists see as an important, and ironically overlooked, factor.

One misconception about neutral theory is that it argues that clear-cut morphological or behavioral differences between species don't have ecological consequences. “Certainly selective differences are operating, but it is interesting to see whether those differences are large enough to overcome stochastic (or random) forces,” Bell says.

“It's not niche or neutral—both things are happening. It's determining the relative importance of the two.”

Mathematical advances have helped sustain interest in neutrality. For example, Rampal Etienne, theoretical ecologist at University of Groningen in the Netherlands, developed a consistent mathematical framework to reliably quantify species richness and dispersal. Often, ecologists have only species abundance data to work with, so it was previously difficult to do anything other than estimate such parameters. In conjunction with the work on density dependence, Hubbell colleague Igor Volkov, physicist at Pennsylvania State University in University Park, Pennsylvania, United States, came up with a formula to calculate the degree to which rare species have a reproductive advantage in a community.

## Niche Fights Back

For the most part, traditional niche ecologists appreciate the challenges that neutral theory presents. “Anyone interested in determining what controls species abundance patterns has to come up with an equally simple theory, or a more complex theory that made better predictions,” says Dave Tilman, ecologist at the University of Minnesota in St. Paul, Minnesota, United States.

Contemporary niche theorists have attempted to distill the niche concept into two components: the requirements of a species to exist in a particular environment as well as the impacts it has on that environment. In doing so, Washington University (St. Louis, Missouri, United States) ecologist Jonathan Chase and University of Texas at Austin (Austin, Texas, United States) ecologist Matthew Leibold, authors of the 2003 book *Ecological Niches—Linking Classical and Contemporary Approaches*, suggest that ecological phenomena can be explained by essentially two characteristic properties that most strongly limit a particular community. Even simplified in such a way, niche theory is not as versatile as neutral theory. However, Tilman points out that neutral theory is limited because it is incapable of predicting which species are rare or abundant. “If we add more complexity to the competition part of the model, we explain more of what we see in nature—why certain species are abundant, which are rare, which change in response to environmental gradients or climate change through time,” Tilman says.

Nonetheless, neutral theory has forced a greater appreciation of the importance of stochastic forces. Inspired by Hubbell's work, Tilman's recent experiments and models incorporate random forces as well as competitive trade-offs among species. In doing so, he and colleagues found that the neutral model fit their data, but it was a niche mechanism—a plant's competitive ability for nitrogen—that predicted more successfully the abundance of species in grassland systems of Minnesota, Kansas, and California. Once mechanisms are clearly defined, ecologists can achieve better predictive power, and implement control measures to limit biodiversity loss.

“When theory, observation, and experiments come together, people take notice.”

Others, however, view neutral theory as a distraction from theoretical developments that incorporate the past hundred years of niche-based work. “Occam's razor should not involve throwing things out that we know to be important,” says Peter Chesson, theoretical ecologist at the University of Arizona at Tucson, Arizona, United States. For example, he takes issue with the importance Hubbell places on random demographic forces. “Demographic stochasticity is quite a weak force when compared with environmental variability and niche differences,” Chesson says, noting that both of those mechanisms are discounted in Hubbell's models. Chesson tackles the co-existence problem by segregating mechanisms into either equalizing or stabilizing functions. “We will solve problems by embracing the complexity of what we know to be true, the heart of our subject, rather than imagining it is far simpler than it really is,” he says.

Simply validating the importance of the niche to diversity is a crucial advance. “People accepted the niche as so intuitive that it hasn't been well tested,” says Peter Adler, plant community ecologist at the National Center for Ecological Analysis and Synthesis in Santa Barbara, California, United States. Indeed, ecologists are prone to prematurely concluding that if niche differences are found among species, trade-offs must exist among the species that allow them to coexist. Jonathon Silvertown, biologist at the Open University in Milton Keynes, United Kingdom, found only 13 papers since 1990 that provide evidence that observed niche differences lead to co-existence of species. “We have to show that these ecological differences cause trade-offs among species and thus influence their coexistence,” says Mark McPeek, ecologist at Dartmouth College, in Hanover, New Hampshire, United States.

## Model Merger

Unifying niche and neutral theories is an obvious next step. Suggested by a number of people, the prevailing notion is that stochastic forces exist on one end of a continuum while deterministic forces occupy the other. Finding any truth that lies between is the challenge. “It's not niche or neutral—both things are happening. It's determining the relative importance of the two,” says Stan Harpole, ecologist at the University of California at Irvine, California, United States. McPeek puts it more simply: “What's missing from each theory is the other one.”

Ironically, the assumption of neutrality will likely be discarded in any merger. “Once you combine theories, you have to throw away the concept of neutrality, but you don't have to throw away other aspects of neutral theory like stochasticity or dispersal,” Etienne says. From a philosophical point of view, the first approximation is the neutral model, then one can insert relevant elements taken from niche theory, he suggests. “We are slowly moving from a very general theoretical mode into more predictive mode,” Chave says. Tilman agrees that ecology is at the precipice of moving from descriptive to more mechanistic and therefore predictive. “When theory, observation, and experiments come together, people take notice,” Tilman says.

Hubbell would like to see that discussion expand to include the realistic expectations from theory. “What's the level of precision and accuracy that theory is required to produce?” Hubbell asks, adding that Newton's theory is approximate to general relativity. Most importantly, Hubbell wants to invigorate the rigor of scientific debate in ecology. “I hope we are becoming more sophisticated in the way we do ecology,” he says. Indeed, many theorists think a merger is unnecessary. McGill, for instance, hopes neutral theory's legacy is the very process of developing mathematics around a set of assumptions so that multiple predictions can be made. “It's very common in other fields, such as physics, to have one theory that makes multiple predictions,” he says. “That is what makes it so powerful.”

While the debate over neutrality will surely continue, Hubbell has been criticized for prematurely calling it a unified theory. Some prominent ecologists, such as University of Tennessee (Knoxville, Tennessee, United States) ecologist Dan Simberloff, do not believe community ecology will identify general principles, let alone a grand unifying theory because ecology is simply too complex. Hubbell points out that neutral theory of community ecology is still in its infancy compared with that of population genetics. While others seek to merge neutral and niche theories, Hubbell is attempting to combine neutral theory with the theory of metabolic scaling, which seeks to explain the consistent relationship between a species' body size and metabolic rate. Using this approach, it may be possible to link energy variation across space and time to the number of individuals of all species in community—thereby further linking physics to ecology.

**Box 1. Neutral Models in Biology Often Spark Controversy**

Neutral models are not new in biology, nor is a subsequent debate about their interpretation. Stephen Hubbell, in fact, modeled his mathematical approach after Japanese molecular geneticist Motoo Kimura's 1968 neutral theory of molecular evolution, which controversially posited that molecular evolution didn't involve natural selection at all. Once advances in gel electrophoresis noted unexpected molecular variation in natural populations, Kimura hypothesized that random genetic drift, not natural selection, could explain the variability found. Although the debate in population genetics continues, the neutral theory Kimura developed helped reconcile the importance of natural selection and genetic drift.

While genetic drift is neutral in population genetics, ecological drift is deemed neutral in the corresponding biodiversity theory. In Hubbell's neutral world, both offspring and immigrants have the same chance of replacing a dead individual in the community. All species experience similar processes. Therefore, speciation exists in a balance with extinction—allowing one to determine whether a species is rare or abundant as a result of random forces.
